# A Modified Mean Stress Criterion for Considering Size Effects on Mode I Fracture Estimation of Rounded-Tip V-Notched Polymeric Specimens

**DOI:** 10.3390/polym14071491

**Published:** 2022-04-06

**Authors:** Ali Reza Torabi, Mahdi Jabbari, Javad Akbardoost, Sergio Cicero

**Affiliations:** 1 Fracture Research Laboratory, Faculty of New Sciences and Technologies, University of Tehran, Tehran 14399-57131, Iran; a_torabi@ut.ac.ir (A.R.T.); mahdi.jabbari@ut.ac.ir (M.J.); 2 Department of Mechanical Engineering, Faculty of Engineering, Kharazmi University, Tehran 15719-14911, Iran; 3 LADICIM (Laboratory of Materials Science and Engineering), E.T.S. de Ingenieros de Caminos, Canales y Puertos, Universidad de Cantabria, Av/Los Castros 44, 39005 Santander, Spain

**Keywords:** polymeric notched specimens, size effect, modified mean stress, notch fracture toughness

## Abstract

The aim of this paper is to assess the size and geometry effects on the mode I notch fracture toughness of polymeric samples containing rounded-tip V-shaped (RV) notches (V-notch with a finite radius at the notch tip). First, using a large number of fracture tests on an RV-notched Brazilian disk and semi-circular bending polymeric samples with four different sizes, the size-dependent values of the notch fracture toughness are obtained. Then, the mean stress criterion is modified for characterizing the size-dependency of notch fracture toughness in polymeric samples. The resulting modified mean stress criterion considers higher order terms of the stress field when calculating the fracture process zone length around the tip of the defect. Additionally, the critical distance *r_c_* is assumed to be associated with the specimen size and a formula containing fitting parameters is utilized for considering this trend of *r_c_*. The comparison between the values of notch fracture toughness obtained from experiments and those predicted by the modified mean stress criterion shows that the suggested approach can provide accurate estimations of size-dependent values of notch fracture toughness in polymeric specimens containing RV notches.

## 1. Introduction

Polymeric materials such as polymethyl-methacrylate (PMMA) and general-purpose polystyrene (GPPS) are widely used in industrial applications due to their good physical and mechanical properties. PMMA (or plexiglass^®^) is an artificial rigid amorphous polymer that can be combined with laser cutting, forming or bending processes. GPPS (also known as crystal polystyrene) is a thermoplastic multi-purpose polymer with brittle behavior and excellent X-ray resistance, low contraction and low production cost. Similar to other polymers, the mechanical properties of both PMMA and GPPS can be improved by adding a suitable percentage of short or long fibers [1,2,3]. Fibers and their orientations affect and control the damage mechanisms of polymers including fiber pull-out, growth of hackles, profuse crazing, energy absorbing, etc. [4,5].

Due to design, lubrication and/or optimization requirements, notch-type defects such as round tip V-shaped (RV) notches may be present in polymeric components. These notches lead to stress concentrations at their vicinity and the load bearing capacity of the corresponding notched components decreases. Therefore, the fracture assessment of notched parts has always been of interest for structural engineers and researchers, particularly when dealing with brittle and quasi-brittle materials such as polymers, given that the fracture process of these materials is generally sudden and catastrophic. 

In this context, for example, Ghadirian et al. [6] investigated the mode I fracture behavior of rock samples weakened by RV and U-shaped notches via a modified form of the point stress criterion. Benvidi et al. [7] inspected the failure of RV-notched parts manufactured from rubber-like materials, utilizing the averaged strain energy density (ASED) criterion. Chen [8] assessed systematically the effectiveness of Filippi’s formulations to define stress fields at the notch tip vicinity of RV notches under pure mode I. Carpinteri et al. [9] probed the brittle fracture of RV-notches utilizing finite fracture mechanics criterion. Ayatollahi and Torabi [10] carried out the assessment of mode I fracture in RV notches by means of the Point Stress (PS) and the MS criteria in polymeric materials. Lazzarin et al. [11] investigated the mixed mode I/II brittle fracture in U- and V-shaped notches applying the ASED criterion. 

A large number of studies associated with the fracture of cracked samples have demonstrated that the fracture behavior of brittle materials, such as polymers, ceramics, concretes and rocks, depends significantly on the size of the sample being analyzed. This has been mainly explained through a local damaged zone around the crack tip called the fracture process zone (FPZ). Bazant [12] suggested tools to determine the quantity of the size effect on the fracture toughness of brittle materials, such as the size effect law (SEL), for both cracked and non-cracked parts. Kim et al. [13] modified Bazant’s SEL by adding an empirical constant. Bazant has published a number of articles analyzing the size effect since 1984, with applications in various quasi-brittle materials (e.g., [14,15,16,17,18,19]). Carpinteri [20] suggested a criterion for the analysis of size effects based on the fractal theory. Hu and coworkers [21,22,23,24,25] proposed and validated a new criterion to describe the size effect according to the length of the FPZ and a boundary effect model. Karihaloo [26] took into account the effect of the softening behavior of quasi-brittle materials in the FPZ by means of the Hillerborg’s model [27] and suggested a criterion to predict the size effect. Cornetti et al. [28] proposed a criterion to consider the size effect on the basis of a combination of energy and strength criteria. Ayatollahi and Akbardoost [29] analyzed the size effect on the fracture toughness of brittle materials, utilizing the modified MTS criterion (MMTS). There are other research papers in association with the size effect on the fracture toughness of cracked samples, such as those studied by Yamachi et al. [30,31], Bazant and Yu [32], Li et al. [33], Khoramishad et al. [34], Ayatollahi et al. [35], Akbardoost and Rastin [36], Çağlar and Şener [37], Gao et al. [38], Akbardoost et al. [39] and Alam et al. [40], among others, demonstrating the great interest in this phenomenon among the research community. 

Contrary to the cracked specimens, there is very limited research in the literature investigating the effect of specimen size on notch fracture resistance (NFR). For example, the size effect on the mixed mode fracture resistance of polymeric U and key-hole notched specimens has been assessed by Torabi et al. [41] using the PS method. Additionally, Torabi et al. [42] used the PS criterion to appraise the size effect on NFR of graphite specimens with various notch types. Di Luzio and Cusatis [43] conducted some fracture tests on rectangular samples with RV notches of various sizes and then utilized the cohesive zone model (CZM) for assessing the onset of fracture in (RV-notched) specimens. Combining the energy release rate approach with an elastic-plastic model, Horn et al. [44] investigated the size and geometry effects on U-notched compact-tension (CT) steel samples. Leguillon et al. [45] predicted the onset of crack growth in RV notches and voids (cavities) considering the influence of the specimen size by using the finite fracture mechanics (FFM) theory. Furthermore, the effect of notch size has been assessed in some studies such as those published by Torabi et al. [46] and Negro et al. [47,48]. 

According to the literature, it can be found that there is no previous analysis estimating the size effect on mode I fracture resistance of RV-notched samples made of PMMA and GPPS. Therefore, this paper aims to analyze the effect of the specimen size on the fracture resistance of polymeric RV-notched samples. Since there are no previous experimental data for polymeric RV-notched specimens with different sizes, 120 RV-notched Brazilian disk (RVNBD) and RV-notched semi-circular bending (RVSCB) samples with different notch radii were prepared and tested. It is shown that the values of the NFT obtained from fracture tests on RVNBD and RVSCB specimens are size-dependent. In order to explain the size dependency of the NFT, a variant of the mean stress criterion, namely MS-Schmidt, used frequently for fracture analyses of notched samples is applied. Additionally, for the first time, the modified mean stress criterion (MMS) is introduced, which considers higher order terms of the stress field when calculating the FPZ length around the defect tip. In both the MS–Schmidt and the MMS criteria, the length of the FPZ is considered to be dependent on the size of the specimen being analyzed. The size-dependent values of the FPZ length are also defined for cracked Brazilian disk (CBD) and cracked semi-circular bending (SCB) specimens with various radii employing a semi-empirical relation. It is finally observed how the suggested criteria successfully predict the mode I NFR of PMMA and GPPS materials considering size and geometry effects.

## 2. Materials and Methods

### 2.1. Material Identification

The materials analyzed in this work are PMMA and GPPS, manufactured by the Cho Chen Ind. Company, Tainan, Taiwan. Following the specifications of the material suppliers, the average ultimate tensile strength (σ_u_), Young’s modulus (E) and Poisson’s ratio (υ) for the PMMA are, respectively, 75.8 MPa, 3.45 GPa and 0.38, while the values of these mechanical properties for GPPS are 45.0 MPa, 3.1 GPa and 0.34, respectively. The tensile strength of PMMA and GPPS is a very important parameter when calculating the FPZ length. Therefore, five dog-bone samples were prepared according to ASTM D638-14 [49] and EN ISO 527 [50] standards for each material and then tested under unidirectional tensile load to acquire a reliable value of σ_u_. These unidirectional tensile tests indicated that the values of 75.8 and 45 MPa reported by supplier companies were accurate, and, thus, these values will be considered in this research. Moreover, the PMMA and GPPS sheets provided in this study were produced by the extrusion technique and their mechanical properties might be different in various directions. Therefore, the isotropy of both PMMA and GPPS materials were demonstrated by testing the tensile samples in two perpendicular directions.

### 2.2. Test Configuration and Preparation of Fracture Specimens

**Figure 1:**
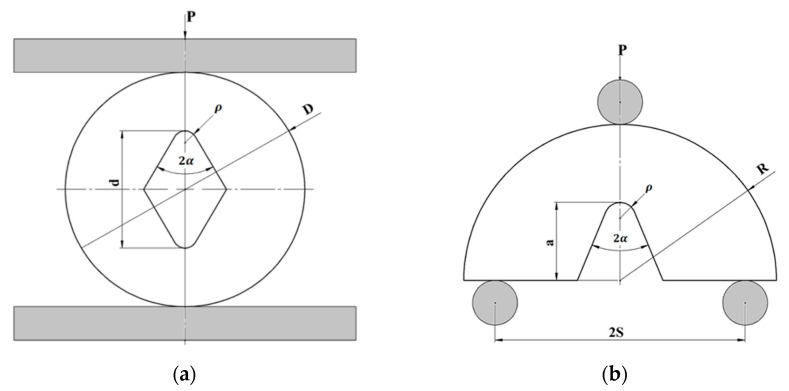
Schematics of the fracture samples: (**a**) RVNBD; (**b**) RVNSCB.

In order to assess the size effect on the NFR of PMMA and GPPS materials, RV-notched Brazilian disk (RVNBD) and RV-notched semi-circular bend (RVSCB) specimens were employed. Figure 1 presents the schematics of RVNBD and RVSCB specimens. RVNBD is a circular disk with radius R = D/2 (D is the diameter of BD sample) and thickness t, having a central rhombic hole in which the large diagonal and notch opening angle are, respectively d = 2a and 2α (a being half of the defect length). The lower and upper corners of the rhombic hole are blunted by the radius of ρ, generating a RV-notch. The RVSCB is a semi-circular disk with a radius of R and thickness of t, in which a RV-notch is generated on the edge of the disk. The angle and length of the resulting RV-notch are 2α and a, respectively. The loading condition in the RVSCB sample is provided by a three-point fixture in which the spam between the supports is 2S. When the direction of the applied load and the bisector line of the RV notch are the same in the RVSCB specimen, and the bottom supports are symmetric relative to this direction, this sample is subjected to pure mode I. Likewise, pure mode I loading is archived in the RVNBD sample by setting the applied load along the bisector line of the RV notch. 

Since the fracture toughness K_Ic_ of PMMA and GPPS materials must be specified for calculating the FPZ length, several cracked Brazilian disk (CBD) and semi-circular bend (SCB) specimens were prepared and tested. The CBD and SCB specimens are similar to the RVNBD and RVSCB samples, except that a sharp crack of length 2a for CBD and length a for SCB is generated in the center of the sample instead of in the rhombic hole and other configurations remain constant. Table 1 gathers the dimensions and loading conditions for all samples. 

**Figure 2:**
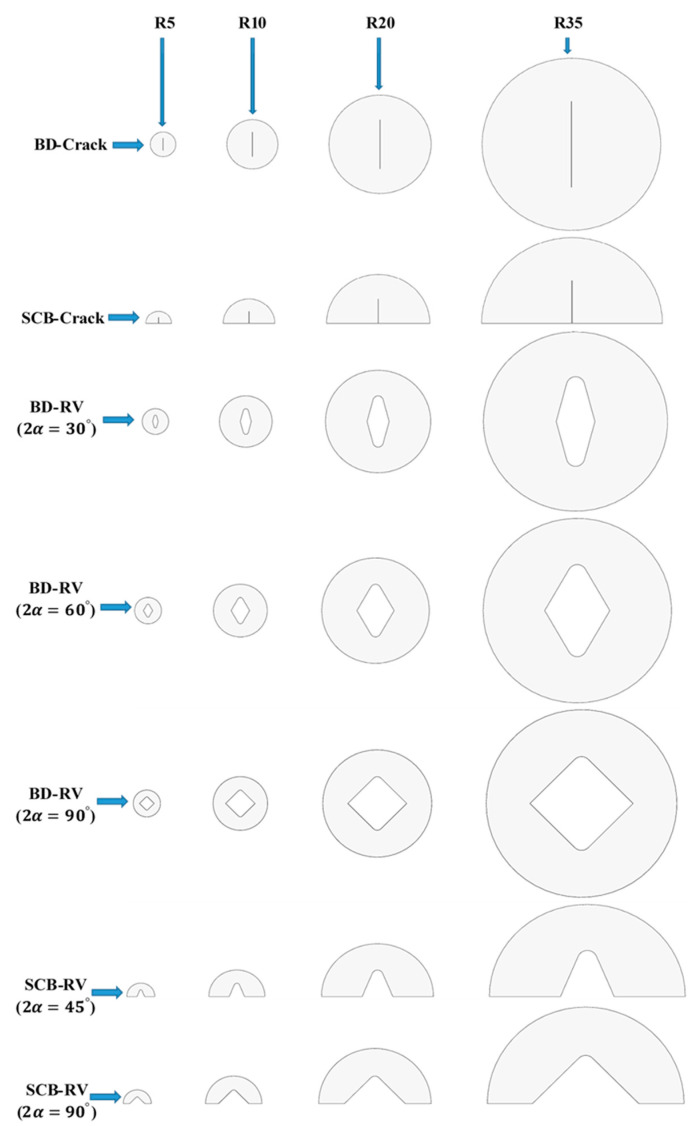
Schematic of scaling on the nominal radius of BD and SCB specimens containing crack and RV notch.

All samples and notches were cut by a waterjet machine from PMMA and GPPS 6 mm thick sheets. The cracks in both CBD and SCB were created by jigsaw with a thickness of 0.2 mm. Then, the generated cracks were sharpened by way of a razor blade with a thickness of 40 µm. Figure 2 shows a schematic of all tested specimens with various sizes. 

### 2.3. Fracture Tests

All notched and cracked samples were tested by using a universal test machine. The tests were carried out by displacement control conditions. The speed of the crosshead was fixed at 0.5 mm/min for all tests, which were continued until final fracture and separation of the two halves of the samples. An LS-20 load cell (2000 kgf), with a maximum error of 0.5% in the range of 2–100% of the nominal capacity was utilized. A load cell with a capacity of 500 kgf was also employed to enhance the accuracy of measurements for smaller specimens with nominal radii R5 and R10 mm.

**Figure 3:**
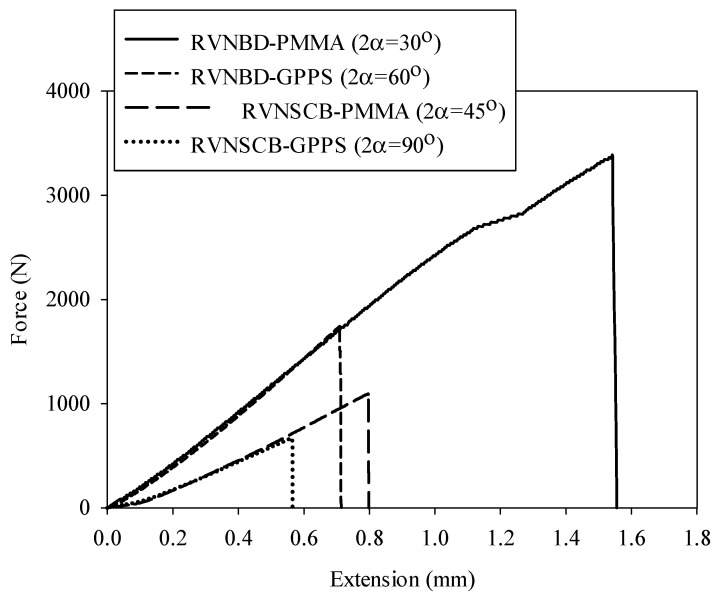
Typical force–displacement curves obtained from the RVNBD and RVSCB polymeric specimens with R = 35 (mm).

The load–displacement curve for each test was recorded, as shown in the example of Figure 3. The load–displacement curves were nearly linear until final fracture, except at the initial moment when a slip occurred between the upper fixture roller and the BD and SCB samples, with no effect on the fracture load. It is noteworthy that experiments on the SCB samples were conducted with two different fixtures due to the different nominal dimensions of SCB specimens. In other words, the two largest specimens (i.e., samples with R = 20 and 35 mm) were loaded using one fixture, while the two smallest ones (i.e., samples with R = 5 and 10 mm) were tested using another fixture with sharper edges. Table 2 and Table 3 show the fracture loads P_f_ in all the tested specimens (cracked and notched, respectively).

**Figure 4:**
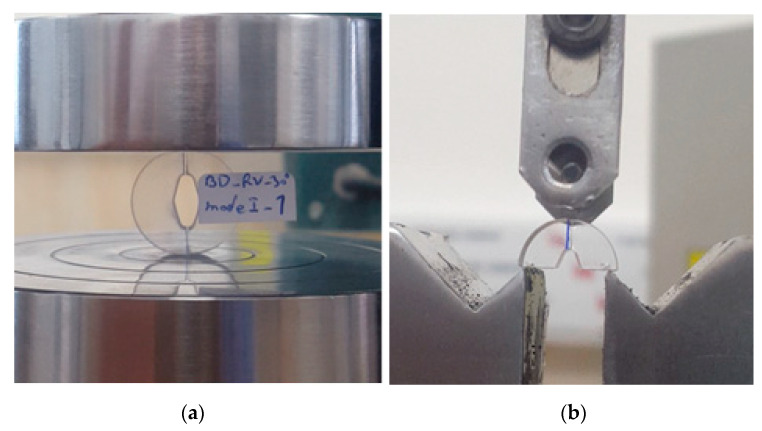
(**a**) RVNBD sample made of PMMA with R = 10 mm and 2α = 30° (mode I loading); (**b**) during test procedure, (**b**) RVSCB sample made of GPPS with R = 5 mm and 2α = 45° (mode I loading).

In addition, Figure 4 shows an example of fractured RVNBD and RVSCB specimens of GPPS and PMMA materials. It can be observed how cracks propagated along the bisector line of the initial notch in RVNBD and RVSCB samples. 

### 2.4. Fracture Toughness and Notch Fracture Toughness Values

The experimental values of the fracture toughness (K_Ic_) of GPPS and PMMA materials may be derived from the CBD and the SCB (cracked) samples. In the case of the SCB samples, K_Ic_ values will be obtained by using the relationship proposed by ISRM standard [51]:

K_Ic_ = Y_I_ ·P_max_ ·(πa)^1/2^/(2Rt), (1)

where S is the half distance between bottom supports, R is the radius of the SCB sample, t is the sample thickness, a is the crack length, P_max_ is the fracture load and Y_I_ is the geometrical factor, which follows Equation (2) [51]:

Y_I_ = −1.297 + 9.516·(S/2R) − [0.47 + 16.457·(S/2R)]·(a/R) + [1.071 + 34.401·(S/2R)·(a/R)^2^, (2)

Concerning the CBD samples, the equation suggested by Akbardoost and Ayatollahi [52] will be used for determining the value of K_Ic_:

K_Ic_ = K_I_* ·P_max_ ·(2πR)^1/2^/(Rt), (3)

where R and t are, respectively, the radius and the thickness of the CBD sample, P_max_ is the fracture load, and K_I_^*^ is the dimensionless geometry factor, equal to 0.2208 when a/R = 0.5 [52]. 

Similarly, notch fracture toughness (K_Ic_^V,^^ρ^) values may be derived from the tests performed on notched specimens. Lazzarin and Filippi [53] proposed the following equation to determine the notch stress intensity factor:

K_I_^V,^^ρ^ = (2π)^1/2^·σ_θθ_(r_0_,0)·r_0_^1−λ1^ /(1 + ω_1_), (4)

where r_0_ is the distance from the origin of the coordinate system to the notch tip [53], λ_1_ is a William’s mode I eigenvalue [53], ω_1_ is an auxiliary parameter depending on the opening angle (α) [53,54], and σ_θθ_(r_0_,0) is the tangential stress at the notch tip, usually obtained from finite element (FE) analyses. The calculation of the notch fracture toughness (K_Ic_^V,^^ρ^) requires, firstly, to develop a (linear-elastic) FE analysis of the corresponding sample and to subject the virtual specimen to the averaged fracture load measured in the experiments, obtaining the tangential stress component along the notch bisector. Then, the value of σ_θθ_(r_0_,0) calculated from FE analysis is substituted into Equation (4). The details of the finite element analyses performed in this research to determine the different values of notch fracture toughness are summarized in Appendix A. 

### 2.5. MS–Schmidt and MMS Criteria for the Analysis of the Size Effect

Two stress-based brittle fracture criteria for considering the size effect are evaluated in this section. These criteria are the mean stress criterion as defined by Schmidt [55] (MS-Schmidt) and the modified mean stress (MMS) criterion. The basis in both cases is the same as that established by the classical mean stress (MS) criterion: the onset of fracture in the notched samples is achieved when the tangential stress averaged over a critical distance (d_c_) attains a critical value of (σ_θθ_)_c_. For brittle materials, the value of (σ_θθ_)_c_ in the MS criterion is assumed to be the material tensile strength σ_u_ [56,57].

Following and simplifying the MS criterion, Ayatollahi and Torabi [6,8] stated that mode I fracture in notched conditions occurs when the notch stress intensity factor (NSIF) K_I_^V,ρ^ reaches the notch fracture toughness K_Ic_^V,ρ^. They also proposed a relation to predict the value of K_Ic_^V,ρ^:

K_Ic_^V,^^ρ^ = (2π)^1/2^·(σ_θθ_)_c_·d_c,V_/{(1/λ_1_)·(d_c,V_^λ1^ − r_0_^λ1^) + [n_θθ_(0)·(d_c,V_^μ1^ − r_0_^μ1^)/(μ_1_·r_0_^(μ1−λ1)^)]}, (5)

According to [8]:

n_θθ_(0) = q·(χ_d1_·(1 + μ_1_) + χ_c1_)/{4·(q − 1)·[1 + λ_1_ + χ_b1_·(1 − λ_1_)]}, (6)

The eigenvalues λ_1_ and μ_1_, as well as the values of χ_b1_, χ_c1_ and χ_d1_, depend on the notch opening angle (2α) [54]. 

The critical distance d_c,V_ is an important parameter in Equation (5). RV notches follow Equation (7):

d_c,V_ = r_0_ + d_c_, (7)

where, again, r_0_ is the distance between the origin of the coordinate axes and the notch tip. According to [54], r_0_ can be acquired as follows:

r_0_ = (π − 2α)/(2π − 2α)·ρ, (8)

d_c_ in Equation (7) is the theoretical length of the fracture process zone (FPZ), based on the MS criterion, and can be obtained from Schmidt’s formula [55] for cracked conditions:

d_c-Schmidt_ = (2/π)·(K_Ic_/σ_u_)^2^ (9)

**Figure 5:**
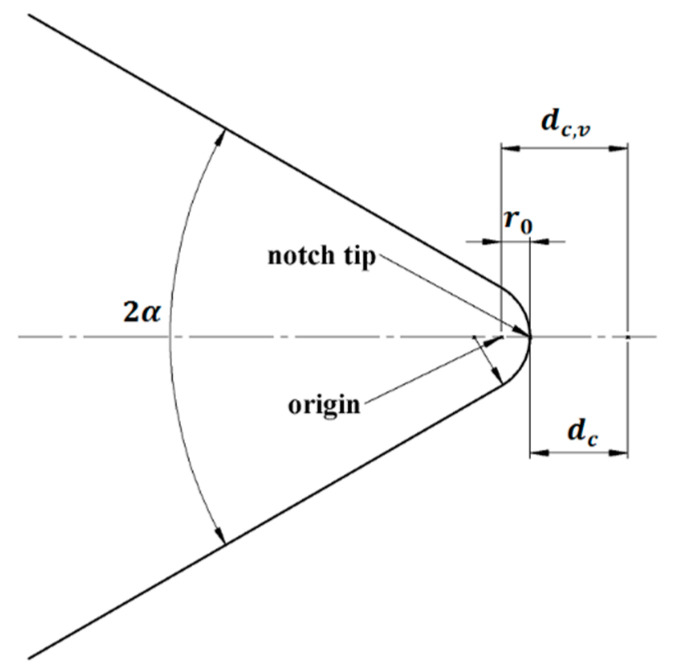
The critical distance d_c,V_ in RV notches according to the MS criterion.

Figure 5 shows a schematic of d_c,V_ in RV notches. The MS criterion, in which the value of d_c_ is obtained from Schmidt’s formula, will be denoted hereafter as the MS–Schmidt criterion.

A second variant of the MS criterion for considering the size effect is the MMS criterion, which considers the higher order terms of the Williams series solution of the stress field when calculating the value of d_c_ in a cracked specimen. More precisely, the tangential stress component is defined by taking into account the first three terms of the Williams series expansion in pure mode I loading [27]:

σ_θθ_ = K_I_·{1 + [3·(A_3_*·r)/(A_1_*·R)]}/(2πr)^1/2^, (10)

where the parameters A_1_* and A_3_* are the dimensionless coefficients of the first and third terms of the Williams series expansion. Then, Equation (11) is used for calculating the tangential stress averaged over the critical distance d_c_:

(1/d_c_)·∫_0_^dc^σ_θθ·_dr = (σ_θθ_)_c_ = σ_u_, (11)

The final equation for d_c_ when using the MMS gives:

d_c-MMS_ = (π/8)·{[R·A_1_*·σ_u_ ∓ ((R·A_1_*·σ_u_)^2^ − (8·A_1_*·A_3_*·R·K_Ic_^2^/π))^1/2^]/(A_3_*·K_Ic_)}^2^, (12)

In Equation (12), the minimum positive value of d_c_ is physically acceptable and will be denoted as d_c-MMS_ for the sake of more clarity. 

Now, d_c,V_ can be calculated according to the new proposed approach as follows:

d_c,V-MMS_ = r_0_ + d_c,MMS_ = (π − 2α)/(2π − 2α)·ρ + d_c,MMS_, (13)

The length of the FPZ depends on the size of the cracked specimen [15,24,27]. Therefore, it can be stated that the parameter d_c_ in both the approaches mentioned above (i.e., d_c-MMS_ and d_c-Schmidt_) is size-dependent. In the present study, the recent approach suggested by Ayatollahi and Akbardoost [27] is used to explore the size effect on the parameter d_c_:

d_c_ = A/(1 + B/R), (14)

in which A and B are fitting parameters that are determined from mode I fracture tests performed in cracked samples with different sizes. Equation (14) may be converted into Equation (15), which is more suitable for linear regression:

1/d_c_ = (1/A) + (1/A)·(B/R), (15)

More details about the calculation of A and B will be described below. 

Now, the size-dependent values of the notch fracture toughness for notched polymeric samples can be predicted by replacing the parameter d_c_ according to the size of the sample from Equations (9) or (12) into Equations (7) or (13) and then substituting the calculated values of d_c,V_ into Equation (5). In the next sections, the notch fracture toughness obtained from experiments will be compared with the predictions derived from the MS criteria (MS–Schmidt’s and MMS).

## 3. Results and Discussion

The fracture toughness (K_Ic_) of GPPS and PMMA materials are easily derived by substituting the fracture loads and sample dimensions listed in Table 1 and Table 2 into Equations (1) and (3), using P_f_ (average values of fracture loads) as P_max_. Table 4 gathers the resulting values of K_Ic_, where it can be observed that the results are dependent on the size of the specimen, with larger values in the larger samples. 

Table 5 gathers the values of the notch fracture toughness obtained for PMMA and GPPS in RVNBD and RVSCB specimens. As in the K_Ic_ values, Table 5 reveals that K_Ic_^V,ρ^ depends on the size of the specimen and becomes larger as the size of the specimen grows.

**Figure 6:**
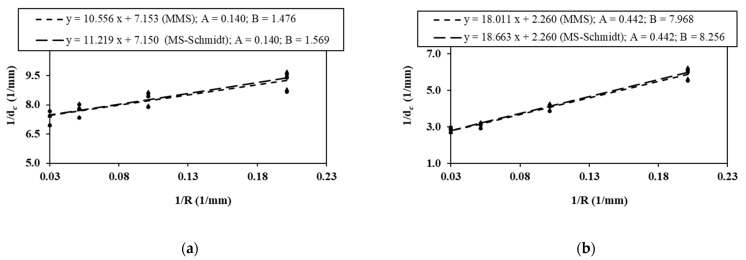

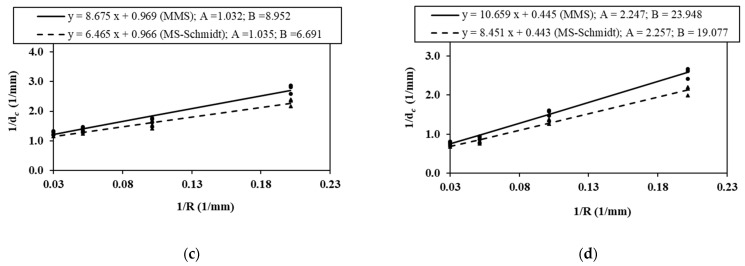
Variations of 1/d_c_ versus 1/R considering both the MS-Schmidt’s and MMS criteria, together with two linear fittings. (**a**) BD specimens made of PMMA; (**b**) BD specimens made of GPPS; (**c**) SCB specimens made of PMMA; (**d**) SCB specimens made of GPPS.

The determination of d_c_ for each combination of sample size, sample type (CBD and SCB specimens) and material (PMMA and GPPS) is the first step in the MS–Schmidt’s and MMS criteria. To do this, the values of K_Ic_ values listed in Table 4 are replaced into Equations (9) and (12). Additionally, the tensile strength σ_u_ in these two equations is considered as 75.8 MPa for PMMA and 45.0 MPa for GPPS. The results are shown in Table 6, where it can be observed again that d_c_ changes by altering the nominal dimension of the sample. To quantify the size-dependency of d_c_, Equation (14) is utilized, while the fitting parameters A and B are determined from a linear regression between the variations of 1/d_c_ versus 1/R, as shown in Figure 6. Once the parameters A and B are calculated for each material and sample, the evolution of d_c_ with the sample size can be found.

**Figure 7:**
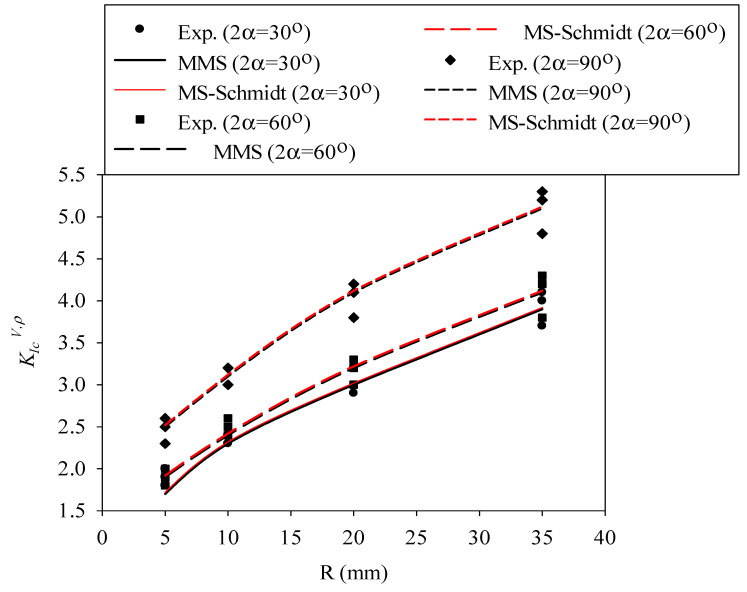
Variation in K_Ic_^V,ρ^ with specimen size for RVNBD specimens made of PMMA. Predictions provided by the MS–Schmidt and the MMS criteria, and experimental data.

**Figure 8:**
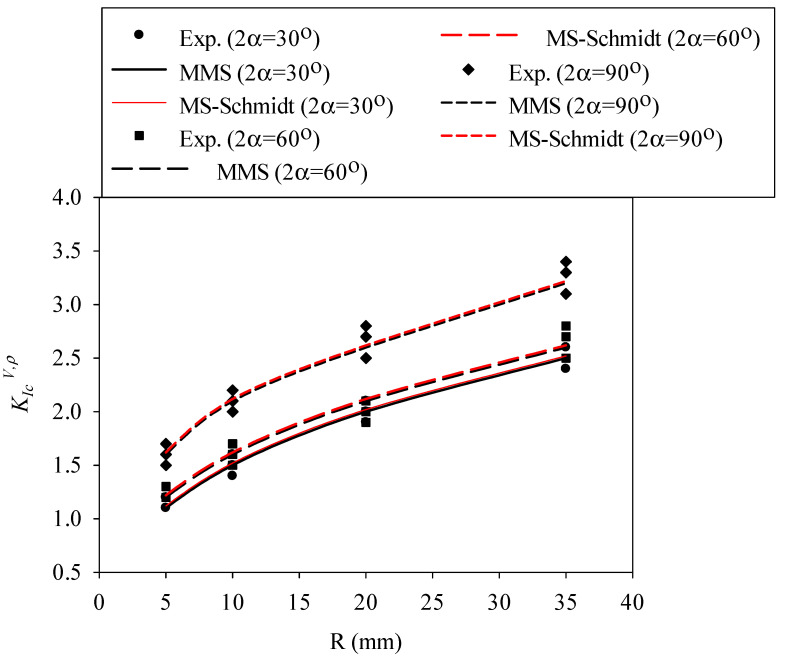
Variation in K_Ic_^V,ρ^ with specimen size for RVNBD specimens made of GPPS. Predictions provided by the MS–Schmidt and the MMS criteria, and experimental data.

**Figure 9:**
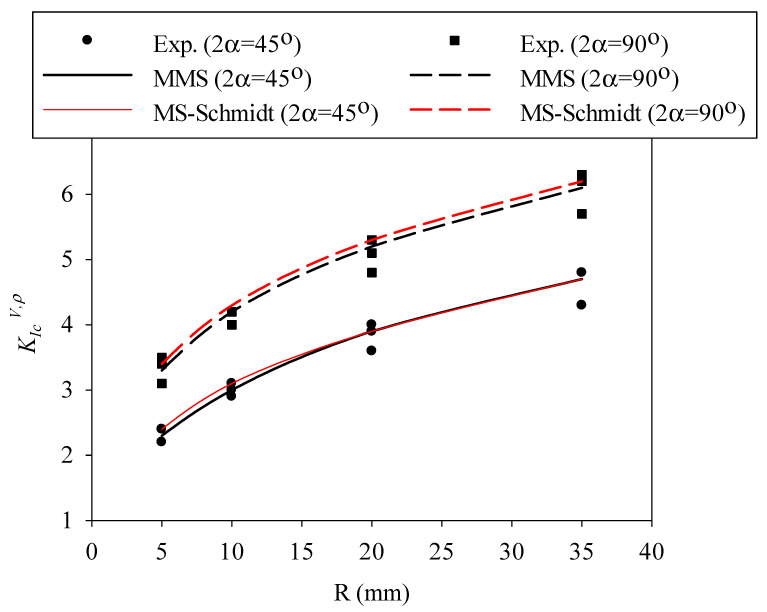
Variation in K_Ic_^V,ρ^ with specimen size for RVNSCB specimens made of PMMA. Predictions provided by the MS–Schmidt and the MMS criteria, and experimental data.

**Figure 10:**
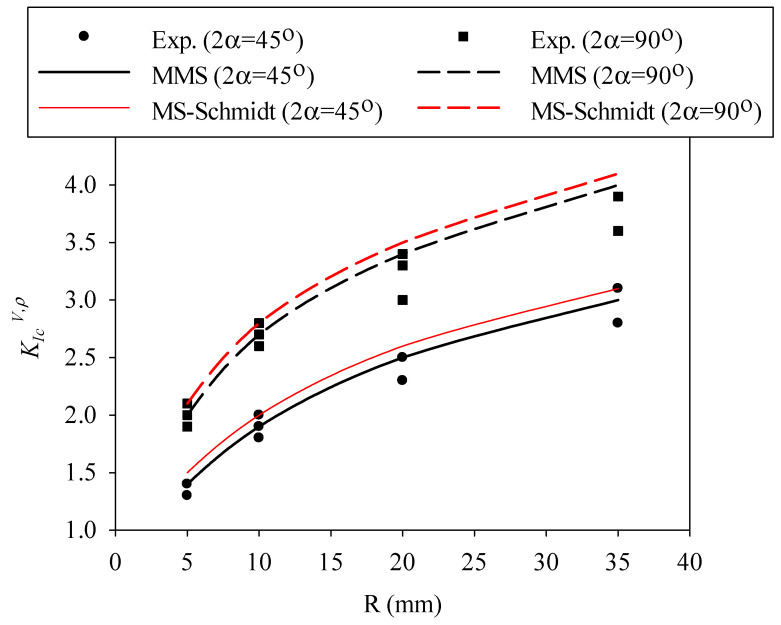
Variation in K_Ic_^V,ρ^ with specimen size for RVNSCB specimens made of GPPS. Predictions provided by the MS–Schmidt and the MMS criteria, and experimental data.

Now, the size-dependent values of d_c_ from Equation (15) for each material are replaced into Equations (7) and (13) in order to determine the corresponding value of d_c,V_ for each sample size. After that, the mode I notch fracture toughness value (K_Ic_^V,ρ^) for each tested specimen is predicted by substituting the parameter d_c,V_ into Equation (5). The variations of K_Ic_^V,ρ^ with the nominal radius (i.e., size) of the specimens are predicted by the MS–Schmidt’s and the MMS criteria and compared with those obtained experimentally from the RVNBD and the RVSCB samples made of PMMA and GPPS, with the results being shown in Figure 7, Figure 8, Figure 9 and Figure 10. According to these figures, it is observable that the MS–Schmidt’s and the MMS criteria provide good estimations of K_Ic_^V,ρ^ in BD and SCB samples made of PMMA and GPPS polymers containing RV notches. 

The results shown in Figure 7, Figure 8, Figure 9 and Figure 10 reveal slight deviations between the experimental data and the theoretical predictions, with such (very moderate) deviations being more significant in RVNSCB specimens. Predictions are accurate for the whole range of sample sizes analyzed in this work and for the two criteria applied in this research. 

In addition, the two criteria provide, in practical terms, the same predictions in RVNBD specimens, and very similar predictions in RVNSCB specimens. Since Equation (9) is simpler than Equation (12) for calculating the parameter d_c_, this formula may be preferred for the design or analysis of notched structures manufactured from PMMA or GPPS materials. Significantly larger differences between the fracture loads predicted by the MS–Schmidt and the MMS criteria can be expected for those engineering materials having generally large FPZs, such as concrete, rock, etc.

## 4. Conclusions

In this study, it was observed that the values of fracture toughness and notch fracture toughness (K_Ic_^V,ρ^) of PMMA and GPPS clearly depend on the size of the samples being tested, in such a way that the fracture resistance of polymeric cracked and notched samples enhances by increasing their nominal size.

In this way, a large number of RV-notched Brazilian disk (RVNBD) and RV-notched semi-circular bending (RVNSCB) specimens containing different notch radii were prepared from PMMA and GPPS sheets and then tested in pure mode I loading.

Two alternative versions of the mean stress (MS) criterion were developed for justifying the size effect on notch fracture resistance K_Ic_^V,ρ^, referred to as the MS–Schmidt criterion and the modified mean stress (MMS) criterion. Thus, two formulations were considered for determining the critical distance d_c_: the first one is the relation proposed by Schmidt and the second one is the formula in which the higher order terms in the Williams series expansion are considered. In both cases, the critical distance depends on the size of the specimen. 

The small discrepancies between the predictions of notch fracture toughness provided both modifications of the MS criterion and the experimental results, observed in all RVNBD and RVNSCB polymeric specimens, demonstrate the ability of both criteria to predict the mode I notch fracture toughness at different scales. It can be stated that in polymeric materials such as PMMA and GPPS, which have a small fracture process zone (FPZ) length, the MS–Schmidt criterion is more efficient due to its combination of accuracy and simplicity. 

## Figures and Tables

**Table 1:** The dimensions and loading conditions for all specimens (all values in mm).

	R	d or a	2S	ρ
CBD	5	5	-	-
	10	10	-	-
	20	20	-	-
	35	35	-	-
SCB	5	2.5	8	-
	10	5	16	-
	20	10	32	-
	35	17.5	56	-
RVNBD $2\alpha =30^{{}^{\circ}},\;60^{{}^{\circ}},\;90^{{}^{\circ}}$	5	5	-	0.5
	10	10	-	1
	20	20	-	2
	35	35	-	3.5
RVSCB $2\alpha =45^{{}^{\circ}},\;90^{{}^{\circ}}$	5	2.5	8	0.5
	10	5	16	1
	20	10	32	2
	35	17.5	56	3.5

**Table 2:** The fracture loads of polymeric (PMMA and GPPS) CBD and SCB samples at various scales (dimensions in mm, load values in N).

Specimen	Material	Specimens Radius, R	Crack Length (a or d)	P_f1_	P_f2_	P_f3_	P_f,average_
CBD	PMMA	5	5	791.0	761.0	708.5	753.5
		10	10	1058.0	1170.5	1148.0	1125.5
		20	20	1679.5	1713.0	1572.0	1654.8
		35	35	2345.0	2277.5	2109.5	2244.0
	GPPS	5	5	524.5	564.0	586.0	558.2
		10	10	972.0	991.0	895.5	952.8
		20	20	1573.5	1473.0	1604.5	1550.3
		35	35	2232.0	2168.0	2008.0	2136.0
SCB (S/R = 0.8)	PMMA	5	2.5	210.0	196.5	209.0	205.2
		10	5	377.3	370.5	339.5	362.4
		20	10	528.8	558.5	551.5	546.3
		35	17.5	773.5	709.7	765.0	749.4
	GPPS	5	2.5	129.5	121.7	130.2	127.1
		10	5	212.5	232.0	236.0	226.8
		20	10	402.0	424.0	419.2	415.1
		35	17.5	596.5	547.5	590.0	578.0

**Table 3:** The fracture loads of polymeric (PMMA and GPPS) RVNBD and RVSCB samples (dimensions in mm, load values in N).

Specimen	Material	2α (deg)	Specimen Radius, R	P_f1_	P_f2_	P_f3_	P_f,average_
RVNBD	PMMA	30	5	593.4	564.3	587.6	581.8
			10	979.7	1083.7	1062.8	1042.0
			20	1981.6	2001.0	1845.6	1942.7
			35	3341.5	3129.6	3308.9	3260.0
		60	5	458.0	435.5	453.5	449.0
			10	795.9	880.6	863.6	846.7
			20	1559.0	1574.4	1452.0	1528.5
			35	2701.4	2530.0	2675.0	2635.5
		90	5	301.3	283.3	303.4	296.0
			10	517.8	564.8	575.3	552.6
			20	1045.4	1057.8	1001.9	1035.0
			35	1816.9	1685.2	1836.4	1779.5
	GPPS	30	5	365.0	343.0	367.5	358.5
			10	623.6	680.0	692.8	665.5
			20	1270.0	1285.2	1217.3	1257.5
			35	2130.3	1975.9	2153.3	2086.3
		60	5	298.0	280.0	300.0	292.7
			10	514.0	560.8	571.2	548.7
			20	988.8	1000.5	947.7	979.0
			35	1741.5	1615.3	1760.3	1705.7
		90	5	192.9	181.4	194.2	189.5
			10	345.0	376.4	383.4	368.3
			20	709.5	718.0	680.0	702.5
			35	1164.7	1080.2	1177.2	1140.7
RVSCB	PMMA	45	5	208.0	195.5	209.4	204.3
			10	346.4	377.8	384.9	369.7
			20	681.0	689.2	652.8	674.3
			35	1110.6	1030.0	1122.6	1087.7
		90	5	219.4	198.8	212.9	210.4
			10	358.4	394.8	383.4	378.9
			20	651.0	688.8	695.6	678.5
			35	1116.3	1072.0	1127.3	1105.2
	GPPS	45	5	120.9	114.9	119.7	118.5
			10	223.2	246.9	242.0	237.4
			20	437.0	441.4	407.1	428.5
			35	701.0	656.6	694.3	684.0
		90	5	128.8	119.7	129.5	126.0
			10	235.0	257.9	256.2	249.7
			20	437.8	440.4	419.6	432.6
			35	702.0	662.7	708.3	691.0

**Table 4:** K_Ic_ values for PMMA and GPPS obtained from CBD and SCB specimens with different sizes (dimensions in mm, load values in N, K_Ic_ in MPam^1/2^).

Specimen	Material	Specimens Radius, R	Crack Length (a or d)	P_f,average_	K_Ic_	Standard Deviation (%)
CBD	PMMA	5	5	753.5	0.983	5.5
		10	10	1125.5	1.038	5.3
		20	20	1654.8	1.079	4.4
		35	35	2244.0	1.106	5.4
	GPPS	5	5	558.2	0.729	5.6
		10	10	952.8	0.879	5.3
		20	20	1550.3	1.011	4.4
		35	35	2136.0	1.053	5.4
SCB (S/R = 0.8)	PMMA	5	2.5	205.2	1.976	3.7
		10	5	362.4	2.468	5.6
		20	10	546.3	2.630	2.8
		35	17.5	749.4	2.727	4.6
	GPPS	5	2.5	127.1	1.224	3.7
		10	5	226.8	1.545	5.5
		20	10	415.1	1.998	2.8
		35	17.5	578.0	2.104	4.6

**Table 5:** K_Ic_^V,ρ^ values for PMMA and GPPS obtained from RVNBD and RVSCB specimens with different sizes (dimensions in mm, load values in N).

Specimen	Material	2α (deg)	Specimen Radius, R	K_Ic_^V,ρ^
RVNBD	PMMA	30	5	1.86 MPa·m^0.4986^
			10	2.36 MPa·m^0.4986^
			20	3.94 MPa·m^0.4986^
			35	3.94 MPa·m^0.4986^
		60	5	1.88 MPa·m^0.4878^
			10	2.48 MPa·m^0.4878^
			20	3.14 MPa·m^0.4878^
			35	4.07 MPa·m^0.4878^
		90	5	2.46 MPa·m^0.4552^
			10	3.14 MPa·m^0.4552^
			20	4.04 MPa·m^0.4552^
			35	5.11 MPa·m^0.4552^
	GPPS	30	5	1.15 MPa·m^0.4986^
			10	1.51 MPa·m^0.4986^
			20	2.01 MPa·m^0.4986^
			35	2.52 MPa·m^0.4986^
		60	5	1.22 MPa·m^0.4878^
			10	1.61 MPa·m^0.4878^
			20	2.01 MPa·m^0.4878^
			35	2.63 MPa·m^0.4878^
		90	5	1.57 MPa·m^0.4552^
			10	2.09 MPa·m^0.4552^
			20	2.63 MPa·m^0.4552^
			35	3.28 MPa·m^0.4552^
RVSCB	PMMA	45	5	2.33 MPa·m^0.4950^
			10	2.97 MPa·m^0.4950^
			20	3.82 MPa·m^0.4950^
			35	4.65 MPa·m^0.4950^
		90	5	3.34 MPa·m^0.4552^
			10	4.12 MPa·m^0.4552^
			20	5.06 MPa·m^0.4552^
			35	6.07 MPa·m^0.4552^
	GPPS	45	5	1.35 MPa·m^0.4950^
			10	1.91 MPa·m^0.4950^
			20	2.43 MPa·m^0.4950^
			35	2.99 MPa·m^0.4950^
		90	5	2.00 MPa·m^0.4552^
			10	2.71 MPa·m^0.4552^
			20	3.22 MPa·m^0.4552^
			35	3.79 MPa·m^0.4552^

**Table 6:** The values of d_c_ for PMMA and GPPS obtained from CBD and SCB samples. K_Ic_ in MPa.m^0.5^, d_c_ values in mm.

Specimen	Material	Specimens Radius, R	K_Ic_	d_c-Schmidt_	d_c-MMS_
CBD	PMMA	5	0.983	0.1070	0.1085
		10	1.038	0.1194	0.1203
		20	1.079	0.1290	0.1295
		35	1.106	0.1356	0.1359
	GPPS	5	0.729	0.1665	0.1702
		10	0.879	0.2427	0.2466
		20	1.011	0.3213	0.3247
		35	1.053	0.3486	0.3509
SCB (S/R = 0.8)	PMMA	5	1.976	0.4326	0.3628
		10	2.468	0.6749	0.5861
		20	2.630	0.7664	0.7049
		35	2.727	0.8240	0.7818
	GPPS	5	1.224	0.4710	0.3896
		10	1.545	0.7504	0.6426
		20	1.998	1.2550	1.0998
		35	2.104	1.3917	1.2763

**Figure A1:**
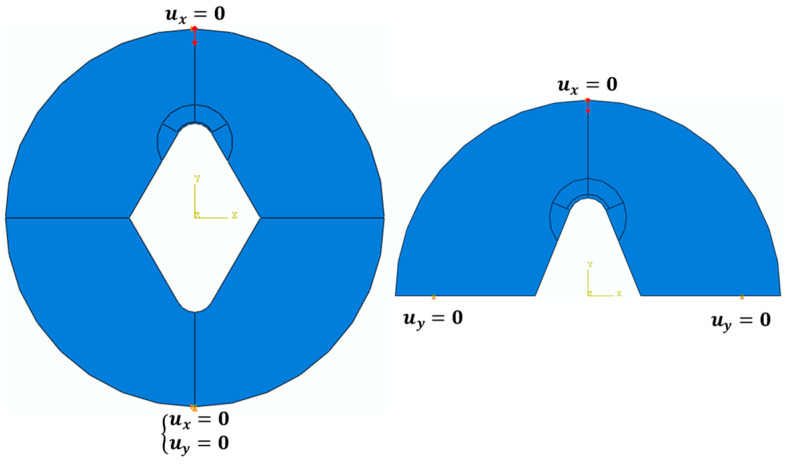
The boundary conditions and partitioning for RVNBD and RVNSCB samples.

**Figure A2:**
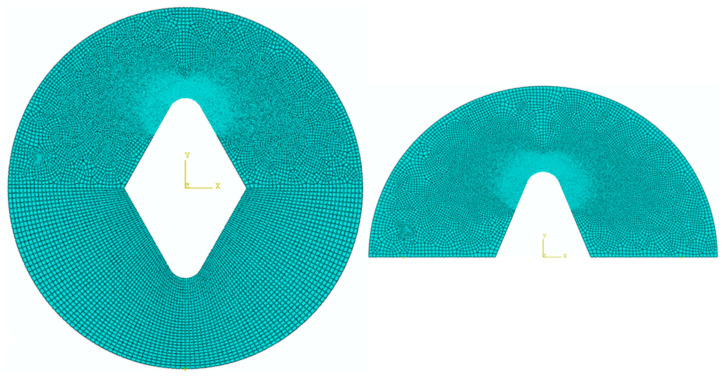
Typical meshing patterns for RVNBD and RVNSCB samples (R5). including RV notch.

**Figure A3:**
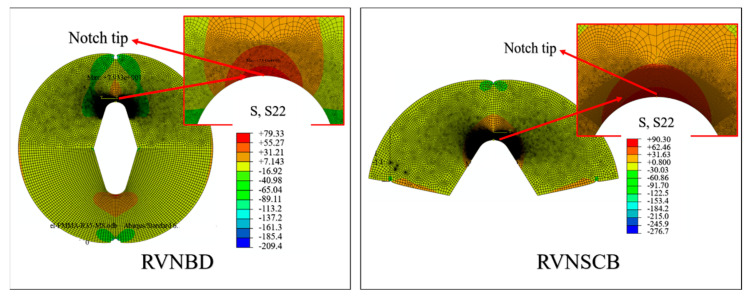
The tangential stress contour around the notch border for RVNBD and RVNSCB specimens of R35.

## Data Availability

The data presented in this study are available on request from the corresponding authors.

## Appendix A

Finite element analyses were performed in pure mode I for determining the critical stress intensity factors (SIFs) and critical notch stress intensity factors (NSIFs) for the cracked and notched polymeric BD and SCB specimens. Specimens were subjected to the corresponding fracture loads. All RVNBD and RVNSCB samples were modeled in two-dimensional form in Abaqus 6.14 code and were meshed by using CPS8R-type elements, which are quadrilateral, 8-nodes, biquadratic, plane stress and reduced integration elements.

It should be noted that the elements around the notch border were very fine with the aim of considering the high stress gradient at the vicinity of these areas. The size of the element for each sample is dependent on its size. For example, the mesh size for samples with a nominal radius of R5 is considered to be 0.01 mm around the notch tip, while its size is 0.1 mm for far-field. For larger specimens, the size of element increases proportionally in both near-field and far-field zones.

Figure A1 shows the partitioning of RVNBD and RVNSCB samples, as well as their boundary and loading conditions. An example of the mesh pattern is also represented in Figure A2 for both specimens. After numerical computations in ABAQUS, the tangential stress component at the notch tip σ_θθ_(r_0_,0) is obtained. Figure A3 displays a typical contour of tangential stress for RVNBD and RVNSCB specimens. The values of σ_θθ_(r_0_,0) extracted from FEA are substituted into Equation (4) for calculating K_I_^V,ρ^.
